# Biodegradation of low-density polyethylene LDPE by marine bacterial strains *Gordonia alkanivorans* PBM1 and PSW1 isolated from Mediterranean Sea, Alexandria, Egypt

**DOI:** 10.1038/s41598-025-96811-z

**Published:** 2025-05-14

**Authors:** Donia M. Wafaa, Mahmoud W. Sadik, Hala F. Eissa, Kareem Tonbol

**Affiliations:** 1 College of Biotechnology, Misr University for Science and Technology, 6th of October City, Egypt; 2 Department of Microbiology, Faculty of Agriculture, Cairo University, Cairo, Egypt; 3 College of Maritime Transport, Arab Academy for Science, Technology, and Maritime Transport, Alexandria, Egypt

**Keywords:** Marine, Plastics, LDPE, Bacteria, Biodegradation, Climate change, Environmental biotechnology, Applied microbiology, Bacteria, Environmental microbiology, Biotechnology, Microbiology

## Abstract

Plastic has become an essential part of daily human activity. Nonetheless, its over-utilization has resulted in environmental accumulation, leading to marine contamination. Biodegradation is the most effective approach for managing synthetic plastic waste. It encompasses various biological processes that depolymerize polymeric compounds into oligomers or monomers that can enter the biogeochemical cycle. Although research on microplastic biodegradation is abundant and increasing, studies on the biodegradation of low-density polyethylene (LDPE) by marine microorganisms remain scarce and underexplored. In the present study, a total of 17 bacterial isolates were isolated from plastic-contaminated sites in Abu Qir Bay, Alexandria, Egypt. Two bacterial strains demonstrated the highest LDPE biodegradation potential and were identified using 16 S rRNA sequencing, exhibiting 100% and 99.87% sequence identity to *Gordonia alkanivorans*. Biodegradation of LDPE was confirmed through dry weight loss, with *G. alkanivorans* strains PSW1 and PBM1 achieving reductions of 0.88 ± 0.658% and 0.66 ± 0.508%, respectively. Biodegradation was further confirmed through the formation of cracks and cavities observed through scanning electron microscopy (SEM). Infrared analysis indicated significant changes in LDPE functional groups and a decrease in the carbonyl index. Biodegradation of LDPE films was also demonstrated through gas chromatography-mass spectrometry (GC/MS) via the release of metabolites, correlating with LDPE utilization. The findings highlight the ability of marine bacteria *G. alkanivorans* strains PSW1 and PBM1 to biodegrade LDPE.

## Introduction

Plastic litter has been found in a wide range of environments, from Mount Everest^1^ to Mariana Trench^2^, as well as in the atmosphere^3^. It has also been discovered in undersea canyons^4^, water columns^5^, beaches^6^, dunes^7^, and mangrove ecosystems^8^. An estimated 12.7 million tons (Mt) of plastic litter enter the world’s oceans each year^9^, and is anticipated to reach 23–37 Mt by 2040^10^. Marine plastic pollution originates from three primary sources—inland, sea-based, and airborne^11^. Among these, rivers play the most significant role in transporting plastics from inland regions to the ocean, contributing to an estimated 1.15–2.41 million metric tons of plastic to the oceans annually^12^.

LDPE, known for its flexibility and moisture resistance^13^, is one of the most common types of plastic waste^14^. Plastic pollution jeopardizes marine ecosystems and undermines the achievement of the Sustainable Development Goals (SDGs) set by the United Nations. The impact of plastic waste on marine environments compromises biodiversity, disrupts food chains, and impairs the health of aquatic ecosystems. Climate change, with its effects such as rising sea temperatures and shifting ocean currents, is expected to impact the distribution and fate of plastic waste^15^. These changes could affect biological carbon pumps, sea ice dynamics, the role of the plastisphere in biogeochemical cycling, and contribute directly to greenhouse gas emissions from microplastics (MP)^16^.

Plastic pollution not only impacts the marine environment but also poses significant risks to human health. It is estimated that humans ingest between 39,000 and 52,000 plastic particles annually^17^. These particles, present in aquatic and terrestrial environments enter the human body through seafood, non-sea food, drinking water, and even atmospheric air^18,19^. MPs can also pass through the epithelial tissue in the digestive system and penetrate cell membranes, leading to adverse effects such as oxidative stress, disruption of energy allocation, cellular damage, and inflammation. Additionally, plastic additives have been linked to severe health issues, including infertility, cancer, and genetic mutations^20^.

**Fig. 1:**
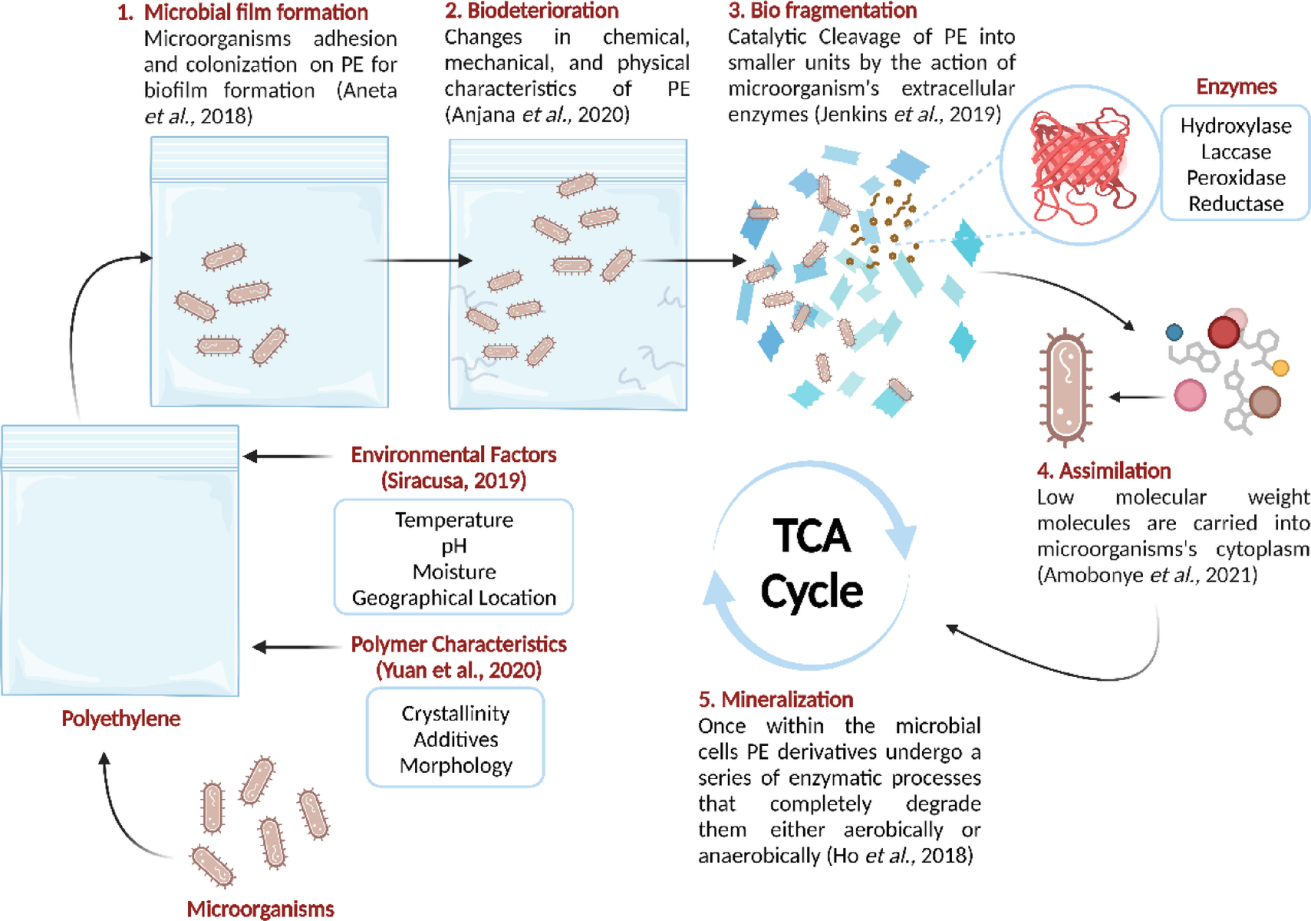
Stages and mechanism of polyethylene biodegradation.

Conventional methods of plastic waste management have proven insufficient to address the magnitude of plastic pollution^21^. Landfilling, the most common method of waste disposal, is restricted in many countries due to environmental and land-use regulations. It contributes to methane emissions, soil and water contamination, and poses risks to animals^22,23^. Incineration, another widely used method, releases toxic gases and heavy metals that harm human health and the environment^24^. Recycling, while beneficial in reducing waste, has its limitations, including the emission of volatile gases that contribute to acid rain, the greenhouse effect, and global warming^25^. Overall, these conventional methods contribute to environmental degradation and fail to provide sustainable, long-term solutions. Meanwhile, there is a growing interest in biological processes like biodegradation—a biotechnological approach that utilizes microorganisms to detoxify and decontaminate plastic waste by breaking down xenobiotics^26^. Microbial plastic biodegradation encompasses many biological activities (Fig. 1) that depolymerize polymeric compounds into oligomers or monomers that can enter the biogeochemical cycle through microbial colonization, biodeterioration, bio fragmentation, assimilation, and mineralization^27^.

Several bacterial species demonstrated significant potential for decomposing plastic polymers and have been isolated from diverse ecological niches, including insect guts^28^, cold marine environments^29^, and seawater^30^. These microorganisms have evolved to metabolize and mineralize plastics, recycling them into chemicals that can be reintegrated into natural cycles. This process is achieved through the action of degradative enzymes which integrate plastic-derived substances into microbial metabolic pathways^31^. Polyethylene-degrading enzymes can be classified into extracellular enzymes, which depolymerize long carbon chains into oligomers, dimers, and monomers^32^, including microbial laccases, peroxidases, lipases, esterases, cutinases^33^, hydroxylases, and reductases^34^. Intracellular enzymes convert these smaller molecules into forms that can be absorbed and utilized by bacteria^35^, through monooxygenases, and dehydrogenases^36^. All of which play critical roles in breaking down plastic polymers.

*Gordonia alkanivorans* have garnered attention in literature for their remarkable ability to biodegrade hydrocarbons^37–39^. These findings underscore the potential of *G. alkanivorans* as a valuable tool in developing sustainable strategies for LDPE biodegradation and environmental remediation. In this study, two strains of *G. alkanivorans* isolated from the plastic-polluted coastal area were investigated for their potential to biodegrade LDPE using analytical techniques such as gas chromatography-mass spectrometry (GC-MS), Fourier-transform infrared spectroscopy (FTIR), X-ray Diffraction (XRD), and scanning electron microscopy (SEM) analysis. This study seeks to address a knowledge gap concerning the existence and potential of plastic-degrading bacteria through the isolation and identification of LDPE biodegrading bacteria from the Mediterranean region and evaluate their degradation efficiency through a comprehensive analysis of the biodegradation process. Ultimately, this study advocates adopting biological methods to achieve a sustainable and plastic-free future.

## Materials and methods

### Study area and sample collection

The Eastern Mediterranean basin is considered the most polluted region in the Mediterranean^40^, Alexandria, Egypt, being a major contributor, releasing an estimated 2209 tons annually of plastic litter in the Mediterranean Sea^41^. Abu-Qir Bay has been identified as a hotspot for plastic pollution, exhibiting the highest sediments and surface water average microplastics (MP) particle concentration, notably LDPE, among all Alexandria beaches^42^.

Three different plastic-contaminated samples (sediments, seawater, and biomass on plastic sheets) were collected and homogenized at Abu-Qir Bay along the Eastern Mediterranean coast, Alexandria, Egypt (31°19′22.1″N–30°03′39.9″ E). Approximately, one kilogram of sediments and several plastic litter sheets were placed in sterile, autoclavable Ziploc bags, while one liter of seawater was collected in autoclavable glass bottles. All samples were stored at 4℃ until further processing.

### Enrichment, isolation, and screening of LDPE biodegrading bacteria

Enrichment for the collected samples was performed using mineral salt medium (MSM) with the mentioned components: 0.7 g/L K_2_HPO_4_⋅3H_2_O, 0.7 g/L KH_2_PO_4_, 0.7 g/L MgSO_4_⋅7H_2_O, 1 g/L NH_4_Cl, 0.005 g/L NaCl, 0.002 g/L FeSO_4_⋅7H_2_O, 0.002 g/L ZnSO_4_⋅7H_2_O and 0.001 g/L MnSO_4_⋅H_2_O_2_^43^. The collected samples—sediments (2 g), biomass (2 g), and seawater (2 ml); were inoculated separately into 250 ml shake flasks (100 ml of MSM and 0.3 g of pre-sterilized LDPE films (3 cm × 3 cm) as the sole carbon source) and incubated in a rotary shaker (150 rpm, 30 °C) for 35 days^44^. Bacterial cultures were then isolated on nutrient agar (NA) plates, incubated at 37 °C for 24 h, and subcultured thrice to ensure purity before being stored at 4 °C for further use^45^. The isolated colonies were individually screened for utilization of LDPE using the pour plate technique on mineral salt agar (MSA)^43,46^. The plates were incubated at 30 °C for 10 days, after which, colonies were enumerated through total viable count^47,48^.

### Bacterial identification

Molecular identification was conducted on the bacterial isolates that demonstrated the highest growth on LDPE-embedded MSM, using the conserved region of the 16 S rRNA gene. The selected isolates were inoculated in 100 ml of nutrient broth (NB) and incubated overnight in a shaker incubator (250 rpm, 37 °C). Genomic DNA was then extracted using the QIAamp^®^ DNA Mini Kit (Qiagen, Germany). The 16 S rRNA gene was amplified using the universal primers sequences (Metabion, Germany): F27 (5′-AGAGTTTGATCMTGGCTCAG-3′), and R1492 (5′-TACGGYTACCTTGTTACGACTT-3′)^49^. The PCR master mix was prepared according to the EmeraldAmp^®^ GT PCR Master Mix (Takara, Japan). The PCR cycling conditions included an initial denaturation at 94 °C for 5 min, 35 cycles of denaturation at 94 °C (30 s) annealing at 56 °C (1 min), extension at 72 °C (1 min), and a final extension at 72 °C (10 min). Amplicons were visualized through a gel documentation system and extracted from the gel using a QIAquick PCR Purification kit (Qiagen, Germany). The purified PCR product was sequenced in forward and reverse directions on an Applied Biosystems 3130 automated DNA Sequencer (ABI, 3130, USA) using a ready reaction big dye Terminator V3.1 cycle sequencing kit. (Perkin-Elmer/Applied Biosystems, Foster City, CA). The obtained gene sequences were analyzed using the BLAST program (http://www.ncbi.nlm.nih.gov/blast).

The phylogenetic analysis was performed using the CLUSTAL W multiple sequence alignment with MEGA11, and the evolutionary history was computed using the distance building-neighbor joining method, with 500 bootstraps. Successively, these sequences were compared with those in the existing GenBank sequence database, and the results were submitted to the NCBI database.

### Inoculum Preparation and biodegradation assay

For inoculum preparation, the selected LDPE-utilizing bacteria were incubated overnight at 37 °C of in Luria-Bertani (LB) broth, and the optical density (OD600nm) was measured using a UV–Vis spectrophotometer. LDPE biodegradation assay of the isolated bacterial strains was carried out as follows: the freshly grown broth culture (1% v/v) was inoculated in 100 ml MSM^50^ with 0.3 g of pre-sterilized 3 cm × 3 cm LDPE sheets as the sole carbon source and incubated at 30 °C at 150 rpm for 30 days^44^. Throughout the experiment the bacterial culture growth rate was monitored through optical density measurements, cell counts/colony-forming units (CFU/ml), and dry weight determination, by collecting 1 ml of bacterial culture and centrifugation at 12,000×*g* (15 min, 25 °C), with the resulting pellet dried overnight (45 °C) before weighing^51^.

### Characterization of LDPE degradation

After 30 days of incubation, LDPE films were repeatedly washed with 2% (v/v) aqueous SDS, then rinsed with distilled water to remove surface impurities before being dried overnight at 45 °C^52^. Polymer biodegradation was assessed at different stages: biodeterioration using SEM, bio-fragmentation using XRD, assimilation through FTIR, and mineralization by GC-MS^53^.

#### LDPE dry weight

The deteriorated LDPE films were regularly removed from the degradation medium and weighed using the formula below to calculate the weight loss percentage^52^.

$$\% \;{\text{Biodegradation }}={\text{ }}\left( {{\text{Initial}}\;{\text{weight }} - {\text{ Final}}\;{\text{weight}}} \right)/{\text{Initial}}\;{\text{weight}}){\text{ }} \times {\text{ }}100$$

#### Gas chromatography-mass spectrometry (GC-MS)

LDPE mineralization was assessed using GCMS (7890B/5977A) (Agilent, USA). The organic contents of the cultivation samples were extracted according to Park and Kim^54^: In brief, 10 ml culture was mixed with 10 ml chloroform in an Erlenmeyer flask, sonicated for 30 min, and left to settle at room temperature for 1 h. The organic phase was transferred to a glass tube in a water bath at 60 °C and then concentrated with nitrogen to 1 ml. Nitrogen served as the carrier gas utilizing a non-polar column (HP-5 (5%-phenyl)-methyl polysiloxane). The oven temperature was maintained at 40 °C for 3 min, then increased to 280 °C at a rate of 10 °C min^−1^, and finally held at 280 °C for 4 min^55^. Soluble products were identified using Agilent Mass Hunter Qualitative Analysis Version B.06.00 in the NIST14 database.

#### Scanning electron microscope (SEM)

Before viewing, LDPE films were flushed with 70% ethanol and dried in a vacuum desiccator for 24 h. The films were then sputter-coated with gold particles and observed under vacuum at a high-resolution SEM on 10,000× and 20,000× magnification^48^.

#### Fourier transform infrared (FTIR)

FTIR analysis was performed to evaluate changes in the structure and functional groups of LDPE film. The frequency range of 4000–400 cm^−1^ was employed with a resolution of 1 cm^−1^ and the relative absorbance intensity of the keto carbonyl bond (KCBI) was assessed using the following formula: Keto Carbonyl Bond Index (KCBI) = I1715/I1465^52^.

#### X-ray diffraction (XRD)

XRD was used to recognize the change in crystallinity related to weight loss. XRD patterns were recorded by a Broker D8 Advanced target Cukα powder diffractometer (λ = 1.5418 Å) between 0 and 80 of 2θ and the crystallinity percentage (%) was calculated by using the following formula: % crystallinity = (area under crystalline peaks/total area under all peaks) × 100%^56^.

### Statistical analysis

The experimental protocols described in the present study were replicated as three independent trials. The variations observed in the values of each trial were tested for statistical significance by one-way analysis of variance (ANOVA) at a probability of *p* < 0.05 using MSTAT-C software. Multiple comparisons of means were analyzed using Tukey’s HSD^57^.

## Results and discussion

### Enrichment, isolation, and selection of LDPE biodegrading bacteria

**Fig. 2:**
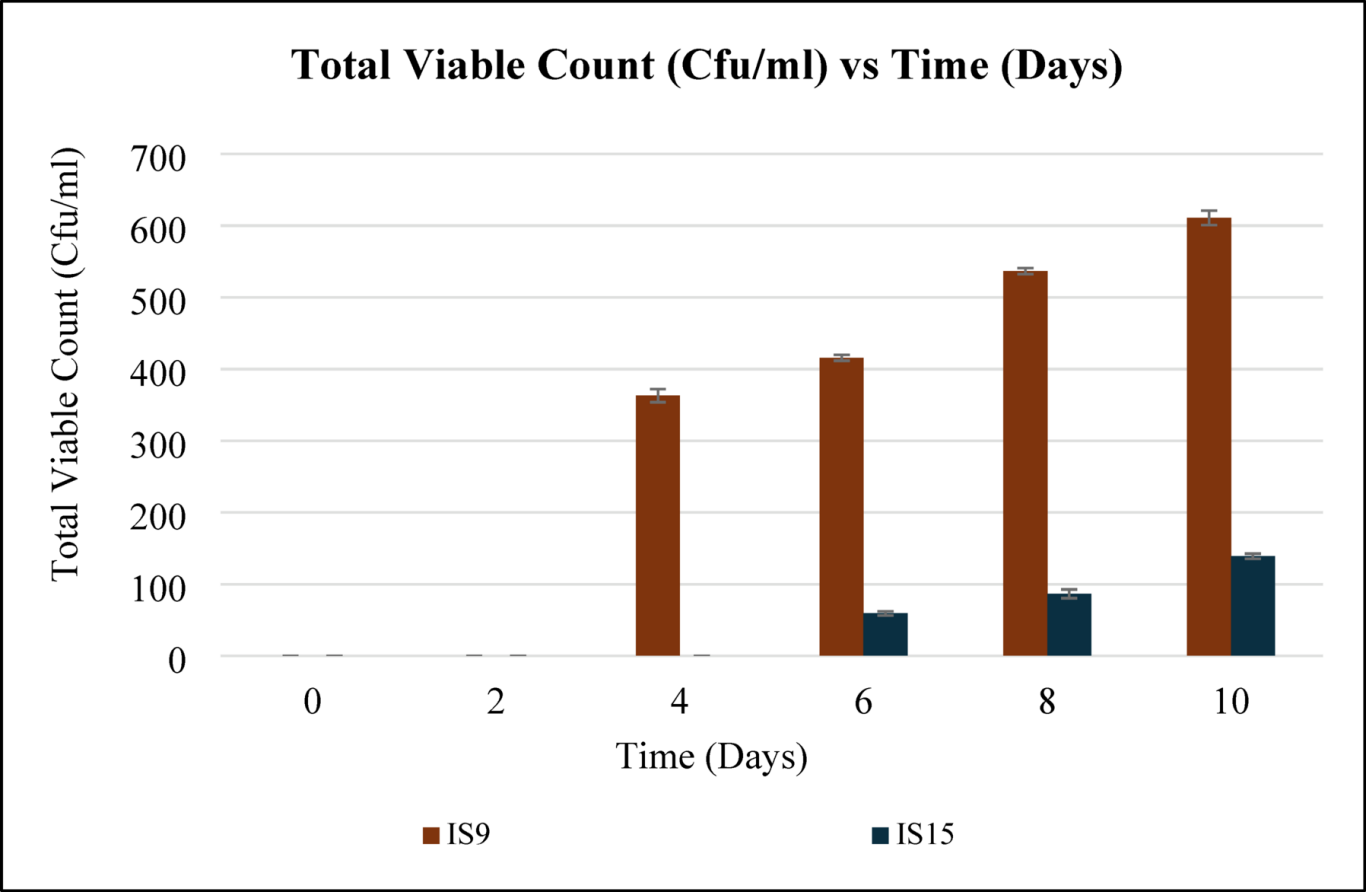
Total viable count (cfu/ml) of the highly selected LDPE biodegrading isolates.

A total of 17 bacterial isolates exhibiting distinctive morphologies capable of growing on LDPE as the sole carbon source were isolated and purified from the enrichment experiment—three from sediments, eight from seawater, and six from biomass on plastic litter. From these isolates, only two isolates, IS9 (seawater) and IS15 (biomass on plastic litter) showed promising growth potential (total viable count) (Fig. 2).

### Bacterial identification

**Fig. 3:**
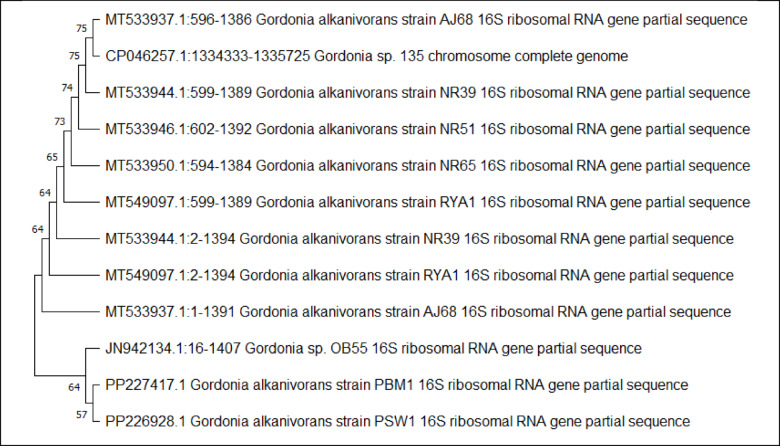
Phylogenetic analysis of *G. alkanivorans* (PP226928.1) and *G. alkanivorans* (PP227417.1) based on 16 S rRNA partial sequencing. Distance-based neighbor-joining tree method based on 16 S rRNA gene sequences and related species constructed using MEGA 11 software at 500 replicates of bootstrap value.

The 16 S rRNA gene sequences of isolates IS9 and IS15 showed 100% and 99.87% identity to *G. alkanivorans*, respectively. Phylogenetic analysis was conducted using MEGA 11 software, employing the neighbor-joining method to construct the evolutionary tree, and the bootstrap analysis confirmed a strong homology with *G. alkanivorans* sequences from BLAST results (Fig. 3). The sequences of both isolates were submitted to GenBank and assigned under the accession numbers PP226928.1 and PP227417.1 for *G. alkanivorans* strain PSW1 (IS9) and PBM1 (IS15), respectively.

*G. alkanivorans* belongs to the phylum Actinobacteria phylum, order Corynebacterial, and family Gordoniaceae. It is a Gram-positive, non-spore-forming bacterium with rhodococci morphology. This aerobic microorganism has an oxidative metabolism, is catalase-positive, arylsulfatase-negative, and susceptible to lysozyme^58^. *G. alkanivorans* is prevalent in both aquatic and terrestrial environments, especially in soils, marine sediments, and wastewater^39^. It is notably prevalent in oil or polycyclic aromatic hydrocarbon-contaminated soils^59,60^ due to its ability to produce extracellular polymeric substances (EPS), which enhance its survival and persistence in hydrocarbon-contaminated environments^61^ while facilitating the degradation of compounds such as n-alkanes, branched alkanes, and cyclic alkanes^37–39^.

### Determination of bacterial growth rate

**Fig. 4:**
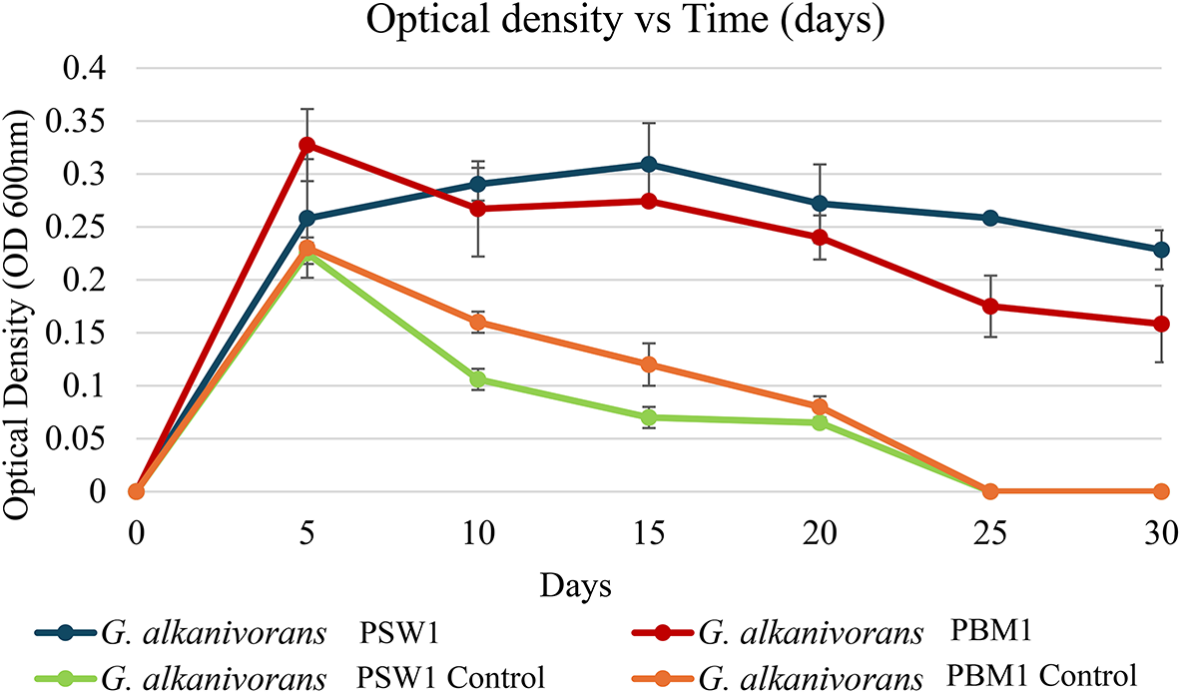
Optical density (OD_600nm_) of *G. alkanivorans* PSW1 and *G. alkanivorans* PBM1 across 30 days incubation.

The biodegradability potential of *G. alkanivorans* PSW1 and PBM1 was assessed using MSM with LDPE films as the sole carbon source. Optical density, total viable count, and biomass dry weight of culture were conducted at specific intervals (0, 5, 10, 15, 20, 25, and 30 days). The growth rate of the two bacterial strains *G. alkanivorans* PSW1 and PBM1 showed significant differences (*p* < 0.05) among the strains. Statistical analysis using one-way ANOVA followed by Tukey’s post hoc test revealed that *G. alkanivorans* PSW1 exhibited the highest growth rate, while *G. alkanivorans* PBM1 showed significantly lower OD600nm values. No significant differences were observed between *G. alkanivorans* PSW1 and *G. alkanivorans* PBM1 after day 10 (*p* > 0.05) (Fig. 4).

According to Khandare et al.^62^, bacterial cultures exposed to LDPE exhibited an increase in optical density and the formation of colloidal suspension, implying bacterial adaptation to LDPE. Eventually, the growth stabilized, consistent with this study in which both strains of *G. alkanivorans* exhibited constant optical density. This stability is presumably due to biofilm growth on the LDPE surface. As bacteria attach to LDPE and form biofilms, growth in the liquid phase of the MSM medium may diminish, resulting in a lower bacterial suspension and, subsequently, a decrease in optical density readings.

**Fig. 5:**
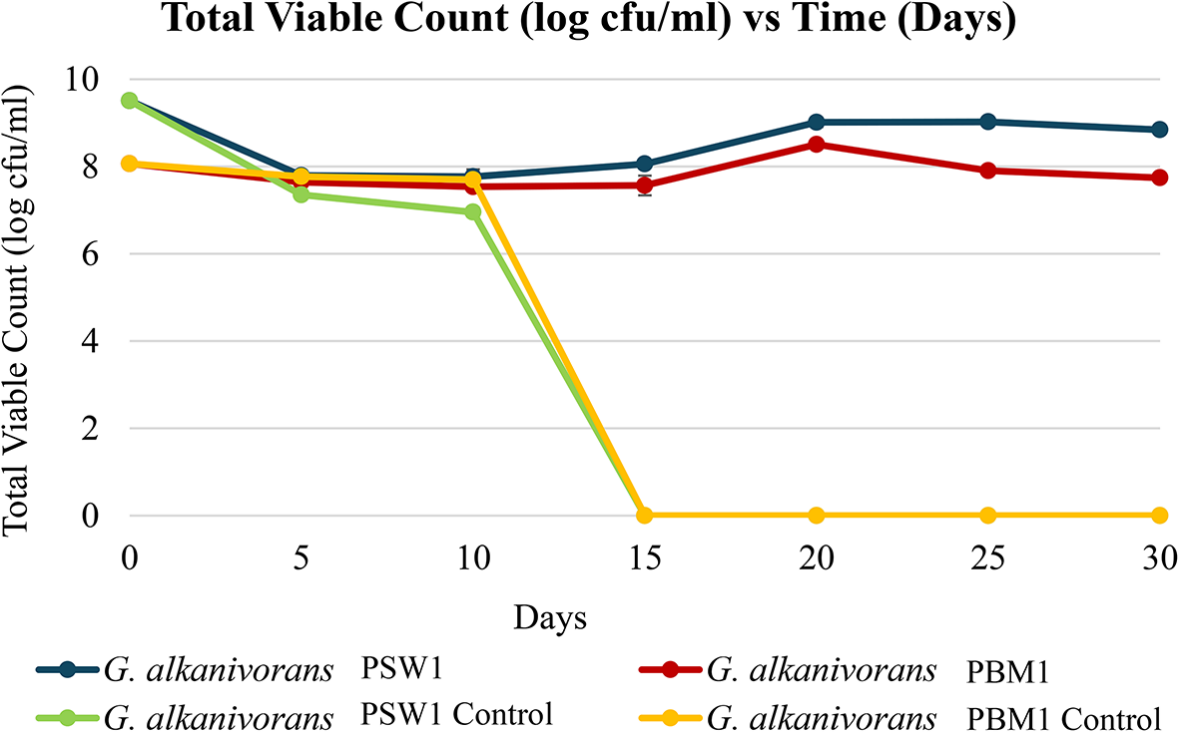
Total viable count (log CFU/ml) of *G. alkanivorans* PSW1 *and G. alkanivorans* PBM1 across 30 days incubation.

In Fig. 5, the total viable count of *G. alkanivorans* PSW1 and PBM1 exhibited significant variations over time (*p* < 0.05). Statistical analysis using one-way ANOVA followed by Tukey’s post hoc test revealed that *G. alkanivorans* PSW1 maintained the highest viable cell count throughout the experiment, showing a slight increase after day 15. Similarly, *G. alkanivorans* PBM1 followed a comparable trend but exhibited a minor decline after day 20. In contrast, the control groups for both strains experienced a sharp decline, with total viable counts dropping to undetectable levels by day 15. This suggests that LDPE degradation played a critical role in sustaining bacterial populations and both strains of *G. alkanivorans* were able to maintain their viability and proliferation through forming of biofilms on LDPE as a matrix of extracellular polymeric substances (EPS) to enhance their survival and persistence^61^.

Zadjelovic et al.^63^ reported minimal growth on non-weathered LDPE, which contrasted with current findings where isolates managed to grow on pristine LDPE without requiring prior weathering. Similarly, Tamnou et al.^64^, reported fluctuations in the total viable count of *Pseudomonas aeruginosa* over time, similar to the observations for *G. alkanivorans* PBM1, where bacterial counts varied depending on their planktonic or biofilm state. In contrast, results for *G. alkanivorans* PSW1 differed from the report from Gupta and Devi^52^, where a total viable count of 8.0 × 10^8^ CFU/ml for *Pseudomonas aeruginosa* strain ISJ14 after 20 days of incubation in MSM with LDPE as sole carbon source, followed by a decline in bacterial counts over the subsequent 60 days.

**Fig. 6:**
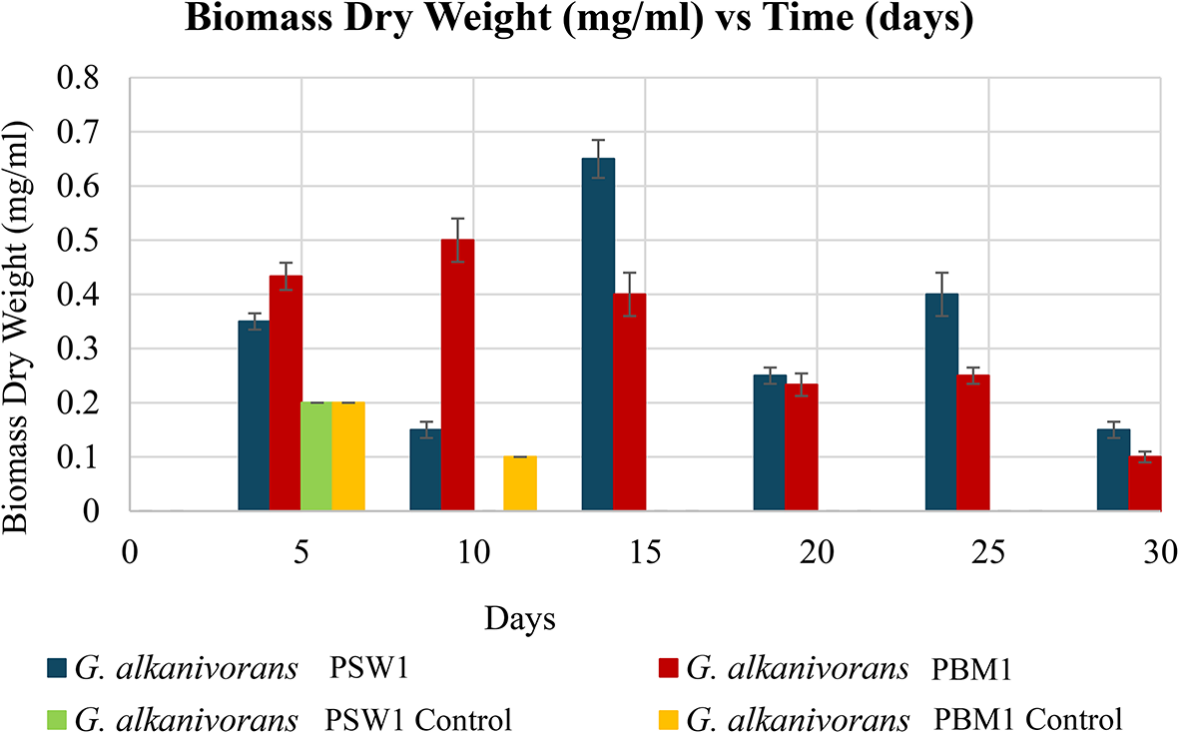
Biomass dry weight (mg/ml) of *G. alkanivorans* PSW1 *and G. alkanivorans* PBM1 across 30 days of incubation.

Furthermore, the fluctuations of biomass dry weight of both isolates correspond to the growth patterns using the optical density values and total viable count (Fig. 6). The biomass dry weight of G. alkanivorans PSW1 and PBM1 exhibited significant differences over time (*p* < 0.05). One-way ANOVA followed by Tukey’s post hoc test confirmed that G. alkanivorans PSW1 achieved the highest biomass accumulation, reaching a peak at day 15, whereas G. alkanivorans PBM1 demonstrated significantly lower biomass values throughout the experiment. The control groups for both strains showed minimal growth, reinforcing the dependence of these bacteria on LDPE as a carbon source. Beyond day 20, no statistically significant differences were observed between G. alkanivorans PSW1 and PBM1 (*p* > 0.05), this could be attributed to the unique properties of LDPE, which serves as a substrate for microbial colonization.

The integration of optical density measurements, total viable count, and biomass dry weight, facilitates a more comprehensive evaluation of microbial colonization and growth on LDPE as the sole carbon source. The mean optical density and total viable count for *G. alkanivorans* PSW1 was greater than that of *G. alkanivorans* PBM1 after 30 days of incubation. Nevertheless, biomass dry weight exhibited no significant variation, indicating that although *G. alkanivorans* PSW1 may possess a superior capacity for growth utilizing LDPE as the sole carbon source; *G. alkanivorans* PBM1, might demonstrate a predilection for LDPE adhesion. This may elucidate its reduced optical density and total viable count since its adherence to the plastic surface could constrain its proliferation in the liquid media.

### Characterization of LDPE degradation

#### LDPE dry weight

**Fig. 7:**
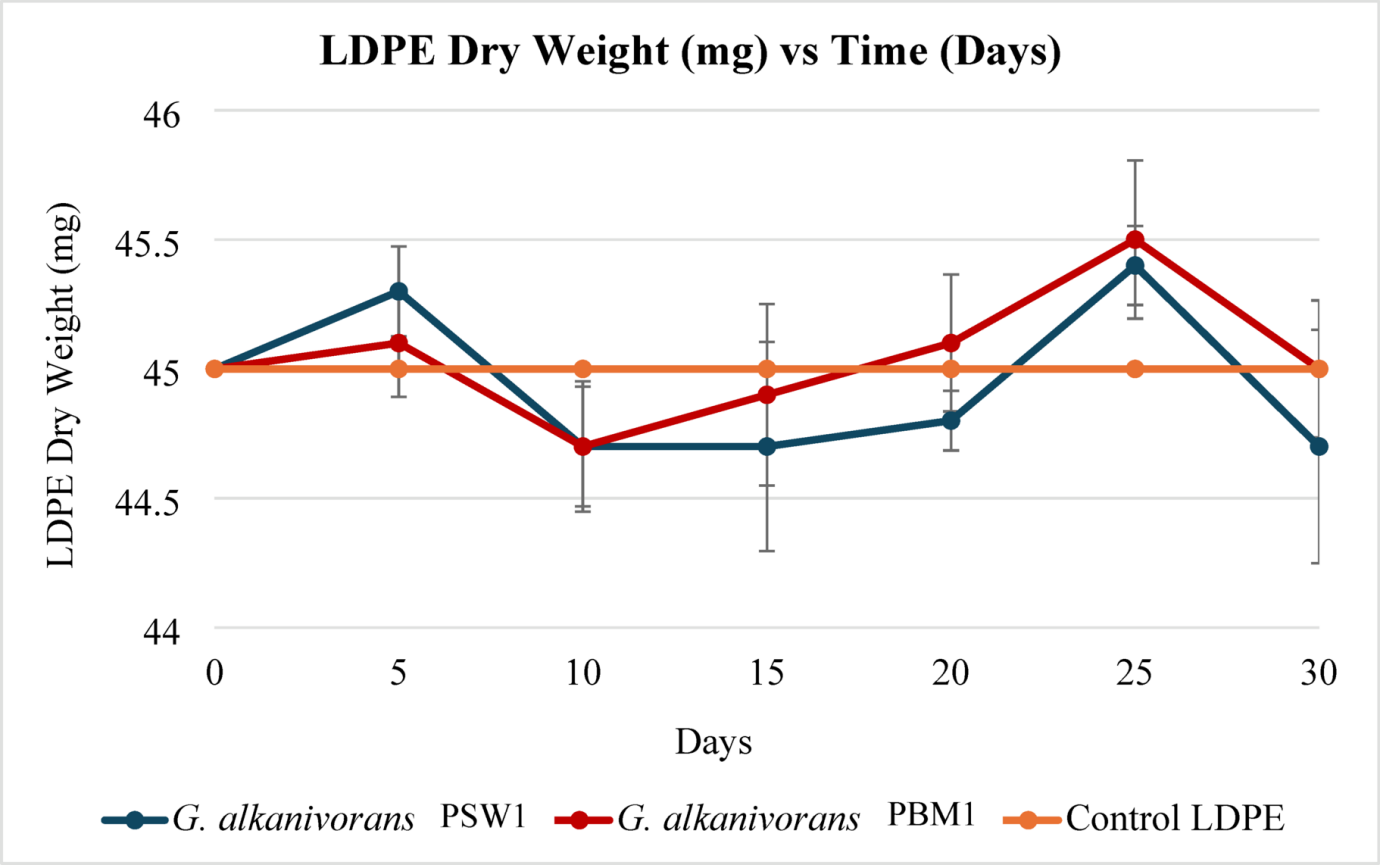
LDPE dry weight of *G. alkanivorans* PSW1 *and G. alkanivorans* PBM1 and control LDPE across 30 days incubation.

The LDPE dry weight exhibited dynamic fluctuations over time, with distinct variations among treatments (Fig. 7). Statistical analysis using one-way ANOVA followed by Tukey’s post hoc test (*p* < 0.05) confirmed significant differences. The control LDPE (untreated with any bacterial culture), remained stable throughout the 30-day incubation period, showing no change in dry weight. In contrast, G. alkanivorans PSW1 and PBM1 demonstrated an initial decline in LDPE dry weight around day 10, followed by a gradual increase, potentially attributable to biofilm formation or bacterial biomass accumulation. By day 25, G. alkanivorans PBM1 exhibited the highest dry weight, likely due to enhanced metabolic activity. However, by day 30, G. alkanivorans PSW1 showed a notable decrease, suggesting potential LDPE degradation, with a total loss of 0.88% ± 0.658 and 0.66% ± 0.508 for G. alkanivorans PSW1 and PBM1, respectively. These findings underscore the complex interplay between bacterial activity and LDPE biodegradation, with statistically significant differences (*p* < 0.05) observed between treated and control samples.

A limited number of research has explored the degradation of unplasticized LDPE film by marine bacteria. This study demonstrated reliable LDPE degradation without additional carbon sources and prior treatments such as UV photo-oxidation, thermal processing or chemical treatments. In contrast, Rong et al.^65^ reported a significant LDPE weight loss of 11.23% by *Rhodococcus* sp. C-2, which may have been enhanced by peptone and yeast extract in the biodegradation medium. These additional nutrients likely facilitated bacterial proliferation and resulted in a higher growth rate compared to *G. alkanivorans* PSW1 and PBM1 in this study. Moreover, *Alcanivorax* sp. 24 exhibited a 0.9% reduction in LDPE weight when grown in an enrichment medium containing hexadecane and succinate, which may have further promoted biodegradation^63^. Conversely, this study showed a comparable LDPE weight reduction of 0.88% in a mineral salt medium devoid of additional carbon sources, highlighting the superior ability of the studied isolates to utilize and degrade LDPE. Under similar biodegradation conditions, *G. alkanivorans* PSW1 achieved a weight loss of 0.88%, surpassing the results reported by Khandare et al.^66^, where marine bacterial isolates, *Marinobacter* sp. H-244 and H-246 and Bacillus subtilis H-248 exhibited maximum weight losses of 0.31%, 0.71%, and 0.35%, respectively. Additionally, Khandare et al.^62^, also reported lower weight losses of LDPE of 0.78%, 0.22%, and 0.46% for marine bacterial isolates *Halomonas* sp. H-255, *Exigobacterium* sp. H-256, and *Alcanivorax* sp. H-265, respectively.

#### Gas chromatography and mass spectroscopy (GC-MS)

**Fig. 8:**
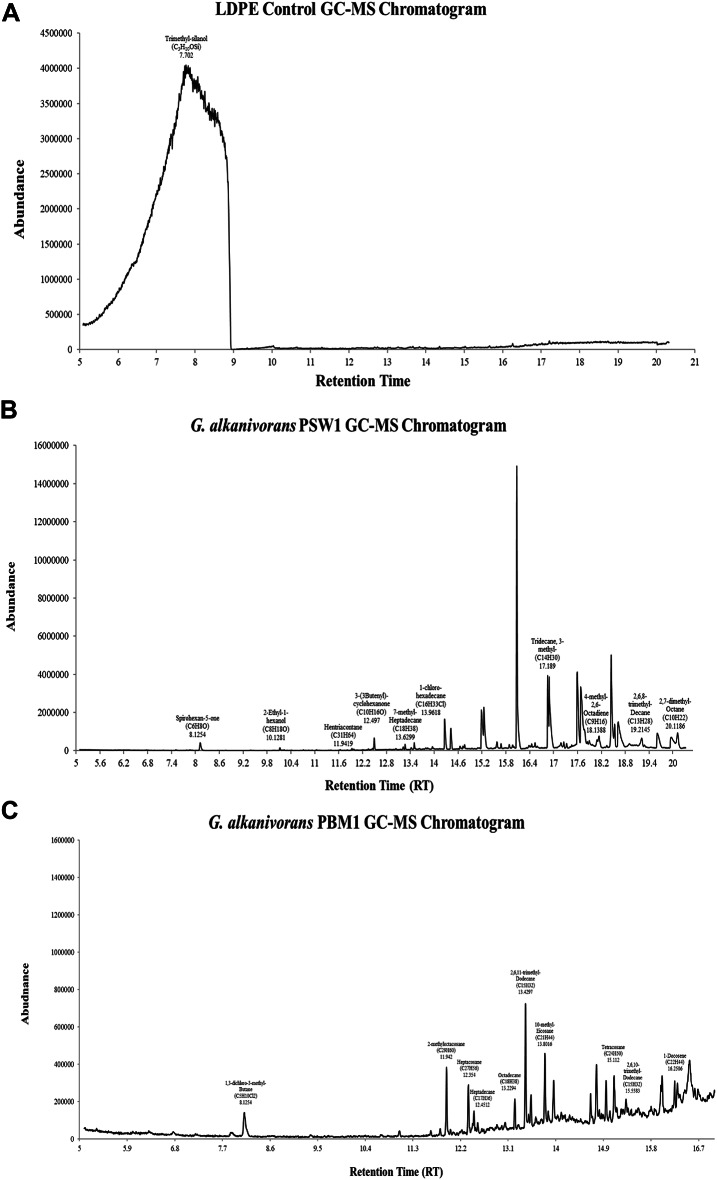
GC-MS chromatogram of (**A**) Control LDPE, (**B**) *G. alkanivorans* PSW1 and (**C**) *G. alkanivorans* PBM1.

GC–MS analysis revealed that the degradation of LDPE by both strains of *G. alkanivorans* produced various compounds, including short-chain alkenes, alcohols, esters, and ketones, confirming the utilization of LDPE film as the sole carbon source. Notably, C5–C31 n-alkanes, are indicative of LDPE biodegradation (Fig. 8b, c). The treatment of LDPE using *G. alkanivorans* PSW1 resulted in the release of 7 alkanes, 2 ketones, and 1 alcohol. While LDPE treated with *G. alkanivorans* PBM1 identified 12 compounds, of which 2 phthalates and 10 alkanes. Consistent with previous findings reported in the literature, similar products have been identified after bacterial incubation with LDPE^43,65,66^. LDPE control was also assessed to determine any leaching products and confirm that the products released were due to bacterial degradation. In the LDPE control, a Peak at 7.702 min was detected that corresponds to trimethyl-silanol (C_3_H_10_OSi) (Fig. 8a), a siloxane that may have been introduced during the experimental process of plastic manufacturing that is widely used as plastic additives^67^. The results indicate that within 30 days, *G. alkanivorans* PSW1 advanced to the hydroxylation phase of LDPE biodegradation, circumventing the preliminary physical and chemical degradation stages. Utilizing a two-stage mechanism for polyethylene degradation, encompassing biodeterioration and biodegradation. Furthermore, *G. alkanivorans* PBM1 demonstrated a promising outcome, based on the degradation products obtained, the biodegradation process may require more time than that needed for *G. alkanivorans* PSW1.

#### Scanning electron microscope (SEM)

**Fig. 9:**
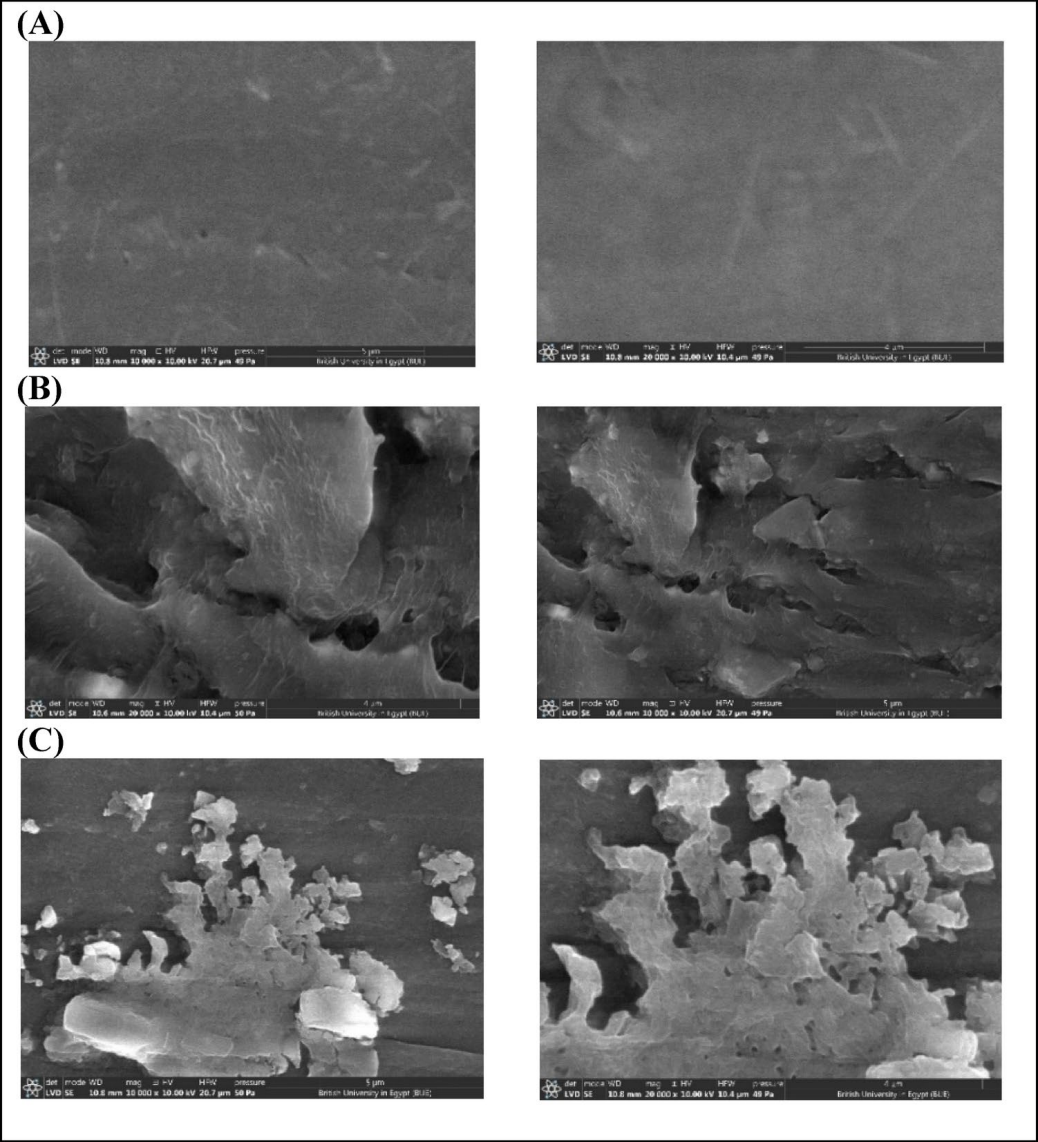
SEM images of LDPE after 30 days of incubation at 10,000× and 20,000× magnification from left to right, respectively, (**A**) control, (**B**) *G. alkanivorans* PBM1, and (**C**) *G. alkanivorans* PSW1.

The control LDPE sheets appeared homogeneous, with no defects or bacterial biofilm observed at high magnification (10,000 and 20,000×) (Fig. 9A). In contrast, LDPE sheets incubated for 30 days with *G. alkanivorans* exhibited significant surface deterioration, including cracks, cavities, pits, and disintegration, along with biofilm formation (Fig. 9B, C). These apparent features confirm the initiation of the biodegradation process, where bacterial activity facilitates the breakdown of polymer chains after extended exposure.

#### X-ray diffraction (XRD)

**Fig. 10:**
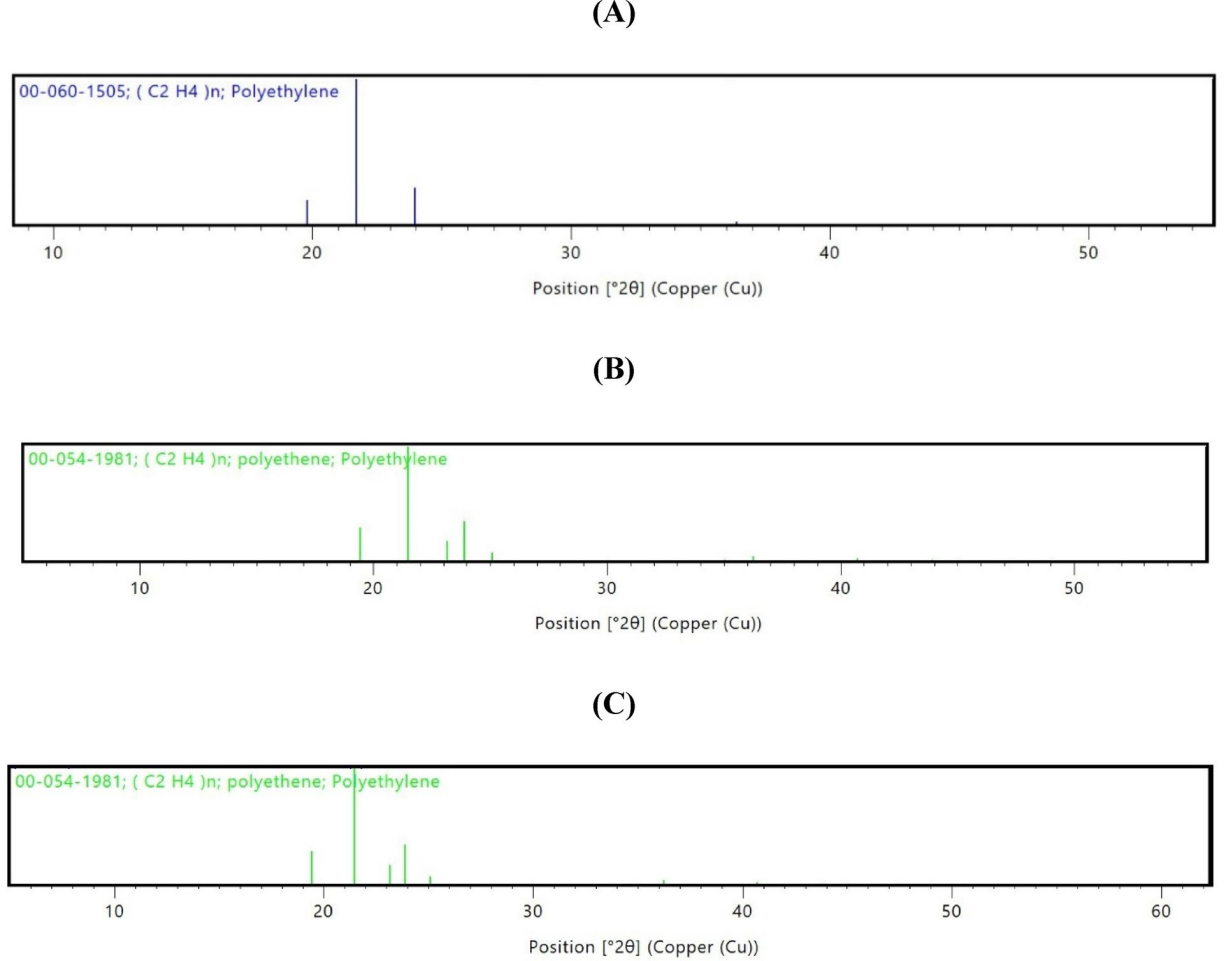
XRD Pattern of (**A**) Control LDPE, (**B**) LDPE treated with *G. alkanivorans* PSW1, and (**C**) LDPE treated with *G. alkanivorans* PBM1.

XRD was used to recognize the change in crystallinity related to LDPE weight loss. In Fig. 10A, the XRD chromatogram of control LDPE exhibited characteristic peaks at 19.6 and 21.3, indicating the amorphous and crystalline portions of LDPE, with intensities of 20.4 and 56.5, respectively. While LDPE treated with *G. alkanivorans* PSW1 displayed amorphous and crystalline peaks at 19.11 and 21.3, with peak intensities of 21.46 and 63.97, respectively (Fig. 10B), and LDPE subjected to treatment with *G. alkanivorans* PBM1exhibited peaks at 19.3 and 21.3, with corresponding intensities of 24.7 and 69.5 for the amorphous and crystalline peaks, respectively (Fig. 10C). The crystalline index for LDPE control, LDPE treated with *G. alkanivorans* PSW1, and LDPE treated with *G. alkanivorans* PBM1 was determined to be 63.89%, 66.45%, and 64.46%, respectively.

According to Maroof et al.^68^, after 90 days of incubation with *B. siamensis*,* B. cereus*,* B. wiedmannii*,* and B. subtilis*, the percentage crystallinity of LDPE films increased. This observation aligns with our findings, suggesting that bacterial activity targets the amorphous regions, leading to an initial rise in crystallinity, which may subsequently decline as bacteria begin to degrade the crystalline portions.

#### Fourier transform infrared (FTIR)

**Fig. 11:**
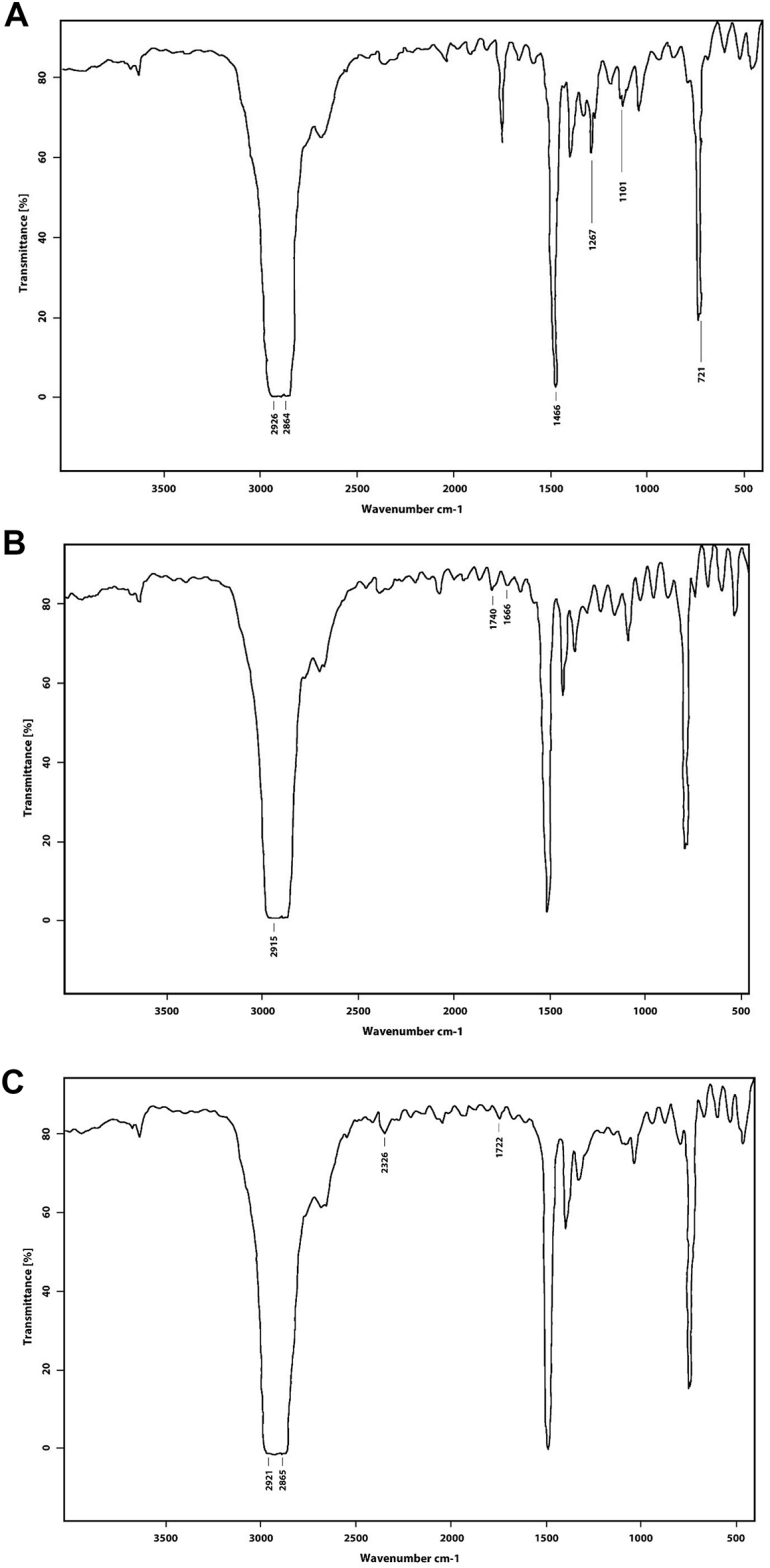
FTIR spectra of LDPE. (**A**) LDPE control, (**B**) LDPE of *G. alkanivorans* PSW1, and (**C**) LDPE of *G. alkanivorans* PBM1.

The transmittance peaks observed in the control LDPE FTIR spectra validate the characteristics of LDPE (Fig. 11A), specifically at 2926 (C-H asymmetric stretch), 2864 (C-H symmetric stretch), 1466 (C-C symmetric bend), and 721 cm^−1^ (CH_2_ rock)^69^. Khandare et al.^66^ reported that the FTIR analysis of bacterial-treated and control LDPE films displayed identical peaks, with two continuous peaks in the spectrum range of 2800–3000 cm^−1^ corresponding to C-H stretching and C–C bond stretching. The C-H stretch signifies the presence of alkane groups, which constitute the backbone of polyethylene. Moreover, according to literature, the FTIR spectra of both control LDPE and bacterial-treated LDPE exhibit peaks at wavenumbers of 2921, 2850, 1473, 1368, and 720 cm^−1^. These peaks correspond to –CH_2_ asymmetrical stretching, –CH_2_ symmetrical stretching, bending deformation, –CH_3_ symmetrical deformation, and rocking deformation, respectively^54,70^.

Meanwhile, compared to the control, (Fig. 11B) showed the same peaks with a shift from 1646, 1727, and 2926 cm^−1^ to 1666, 1740, and 2915 cm^−1^, respectively. While in (Fig. 11C) a shift in peaks 1727, 2864, and 2926 cm^−1^ to 1722, 2865, and 2921 cm^−1^, was observed respectively and a new peak appeared at 2326 cm^−1^ that was not in LDPE control indicating an alteration in alkyne groups^71^. Compared to the control, 1101 and 1267 cm^−1^ peaks disappeared in LDPE treated with both *G. alkanivorans* strains, indicating an increase in C-O stretching, ergo causing polymer decomposition by transforming the carbonyl group to alcohol^66^.

This study is consistent with previous findings reported in the literature indicating a shift in peaks between LDPE control and bacterial-treated LDPE, is influenced by the incubation period, the type of bacteria employed in each study, and their respective biodegradation mechanisms^62,71–74^. The carbonyl bond index (CBI) was calculated to assess the extent of biodegradation, thereby validating structural changes and other variations resulting from the bacterial degradation of LDPE. The CBI (CBI = A1727/1465) for LDPE control, LDPE treated with *G. alkanivorans* PSW1, and LDPE treated with *G. alkanivorans* PBM1 was 0.11, 0.047, and 0.044, respectively, indicating a twofold decrease. The reduction in CBI of LDPE treated with both *G. alkanivorans* strains in comparison to the LDPE control, suggests that the bacteria have metabolized the polymers over a 30-day incubation period.

According to Joshi et al.^71^, minimal differences were observed in CBI of LDPE after bacterial 30 days of incubation compared to control. However, on day 120, a nearly twofold decrease was found. On the contrary, Bhelose and Malik^72^, noted an increase in CBI of bacterial-treated LDPE from control, suggesting the formation of carbonyl C=O and vinyl C=C groups and alterations in the polymer’s structure and crystallinity. Furthermore, CBI was diminished following a period of 180 days; the drop-in CBI supports the idea that microbes have used oxidized polymers. In this study, both *G. alkanivorans* strains demonstrated a decreased CBI, indicating the breakdown of LDPE. This reduction suggests the cleavage of ester linkages and the incorporation of oxygen-containing functional groups into the polymer structure, facilitating the conversion of LDPE into compounds more susceptible to microbial attack and subsequent degradation.

## Conclusion and future recommendations

This study described the first report of *G. alkanivorans* as an effective LDPE degrader, contributing new insights to the area of microplastic biodegradation. The bacterial isolates demonstrated the ability to utilize LDPE as the sole carbon source without additional pre-treatment. Biodegradation of LDPE film was characterized by weight loss, FTIR, XRD, SEM, and GC-MS analysis. In an incubation period of 30 days, weight loss of 0.88 ± 0.658% and 0.66 ± 0.508% was observed for *G. alkanivorans* PSW1 and PBM1, respectively. SEM analysis revealed changes in treated LDPE film in the form of cracks, pits, and increased surface roughness. XRD analysis showed increased crystallinity, while FTIR revealed chemical transformations, bond scission, and functional group modifications generally associated with biofilm formation and biodegradation activity. Further, the GC-MS confirmed the utilization of LDPE film after 30 days of incubation through the presence of short-chain alkanes, ketones, and alcohol. These findings underscore the potential of *G. alkanivorans* in LDPE biodegradation, offering a promising solution for mitigating plastic pollution. However, plastic degradation is a complex and time-intensive process, and the marine environment poses additional challenges for LDPE biodegradation, such as fluctuating temperatures, salinity, nutrient availability, and pH levels. These environmental factors can significantly impact the growth and biodegradation activity of bacterial cultures.

The inability of marine bacteria to fully mineralize LDPE is a significant limitation. The formation of low-molecular-weight oxidized fragments and other intermediate degradation products during the partial breakdown of LDPE by marine bacteria could potentially accumulate in the marine environment and have toxic or ecotoxicological effects on marine organisms, potentially disrupting the natural balance and functioning of marine ecosystems. To mitigate these potential environmental impacts, it is crucial to continue research focused on enhancing the complete mineralization of LDPE by marine bacteria, as well as understanding the fate and effects of the intermediate degradation products in the marine ecosystem through ecotoxicological assessments and microbial community analysis. Climate models can be useful tools in understanding the potential impact of plastic pollution on the environment through observing the dispersal and transport of plastic pollution by studying ocean currents, wind patterns, sea surface temperature, ocean acidification, and extreme weather events, to gain valuable insights into the potential long-term consequences of plastic pollution under different climate change scenarios. In marine ecosystems, bacteria rarely exist as isolated entities, but rather as part of complex microbial communities. These communities often engage in synergistic interactions, where different bacterial species work together to degrade complex compounds. Those microbial communities are always associated with other marine organisms, such as sponges, corals, and algae, that could be a reservoir for new bacterial strains that possess enzymes capable of breaking down LDPE, and as the marine ecosystems are intricately connected through biogeochemical cycles, the degradation of LDPE by bacteria results in the release of breakdown products, which can serve as nutrients for other organisms in the marine food web. By studying these interactions, we can identify cooperative relationships that enhance LDPE degradation and potentially develop strategies to harness these interactions for efficient biodegradation and understand the fate and impact of these breakdown products in marine ecosystems to assess the overall ecological consequences of LDPE biodegradation and its potential benefits for nutrient cycling and ecosystem functioning. Moreover, by leveraging genomics, bioinformatics, and molecular docking, we could gain a deeper understanding of the genetic basis of LDPE degradation, identify new enzymes or bacteria with LDPE-degrading potential, and facilitate the design of more efficient biodegradation strategies through bacterial encapsulation. These integrated approaches contribute to more accurate predictions, effective mitigation strategies, and informed decision-making regarding plastic pollution management and climate change adaptation.

## Data Availability

The datasets used and/or analyzed during the current study are available from the corresponding author on reasonable request.
